# Massachusetts Dental Society

**Published:** 1902-05

**Authors:** 

**Affiliations:** Massachusetts Dental Society


### MASSACHUSETTS DENTAL SOCIETY.

(Continued from page 272.)

*Friday, June 6, 1901—Morning Session.*

At the morning session on Friday the following clinics were successfully given:

Victor H. Jackson, M.D., D.D.S., New York City, Demonstrating the Jackson System of correcting Irregularities of the Teeth.

Chas. A. Meeker, D.D.S., Newark, N. J., Bleaching of Teeth with Pyrozone.

Charles C. Patten, D.D.S., Boston, Mass., Porcelain Inlays with Hammond Electric Furnace, illustrating it as a Practical Method.

Dwight M. Clapp, D.M.D., Boston, Mass., Combination Fillings.

Dr. T. D. Shumway, Plymouth, Mass., Tin and Gold Filling by Affinity.

Lawrence W. Baker, D.M.D., Boston, Mass., " A few Ideas."

Dr. Frank L. Marshall, Boston, Mass., Staple Crowns.

Arthur A. Libby, D.M.D., Boston, Mass., A Demonstration of Burnishing Gold.

Dr. Charles H. Gerrish, Exeter, N. H., Non-Cohesive Gold-Foil.

George C. Ainsworth, D.D.S., Boston, Mass., Tin as a Filling-Material.

Dr. Gustave P. Wiksell, Boston, Mass. Repair of Crowns and Bridges in the Mouth.

Edwin C. Blaisdell, D.M.D., Portsmouth, N. H., Non-Cohesive Gold.

Dr. Julius F. Hovestadt, Boston, Mass., Soldering Porcelain Facings into Gold Crowns without investing.

Robert T. Moffatt, D.M.D., Boston, Mass., Carving and baking Teeth, using the Hammond Electric Furnace.

*Friday, June 6, 1901—Afternoon Session.*

The meeting was called to order by the President at 2.30 P.M.

The following paper, on the subject " Good of the Order," was read by Dr. Chas. H. Gerrish, of Exeter, N. H.:

What I have to say does not possess the dignity of a paper, for it has not been written out. I shall appeal largely to the younger men of the profession, the young man who has his future before him, who is standing upon the threshold of the profession, who has gone into it not for the money he will get, but with the lofty, high, and noble purpose of doing good to mankind, and who thus makes use of the grandest opportunities of his life. I feel that I have been highly honored in being asked to give a clinic and a paper too.

Now, I am going to ask you one question: What do you think ought to be charged patients for cleaning their teeth? I do not want you to answer it until I give you my idea of what cleaning teeth means. "That is what we call it in the country." In the city you sometimes dignify it by the name of treatment. When a patient comes into the office and asks, "What will you charge me for cleaning my teeth?" what answer will you give him? Some thirty years ago I had a patient of Dr. Riggs's, of Connecticut, come to me with his child, a son who was in the academy. He said to me, "I am very particular to have you do this cleaning as I wish it done." I took a post-graduate course right then and there. I treated the teeth under the direction of Dr. Riggs, and I secured an insight into what that meant. That plan is to treat the teeth of the young as soon as they emerge from the gum. When a child comes to you with his second teeth just beginning to erupt, it is your business to begin at once to take off the decayed enamel. I have no respect for the enamel as a preserver of teeth. It is a superstition indulged in by most people that the enamel keeps the teeth. Why is the dental profession increasing in numbers? The greatest factor in the answer to that question is this belief that enamel preserves teeth. I take it off instantly, as soon as it shows itself defective. I use anything to do it with that will smooth and polish all the surface of the teeth.

The instant you see a white particle appear on the child's tooth, you say to the parent that tooth will decay in a very short time. You wait for that decay to go on, and it goes on. I wait for nothing, but I begin then and there to polish and smooth it off. I had a case which I was very much interested in a long time ago. This case, I may say, interested me most particularly. I had a child brought to my office who had pits running across the teeth. What shall be done with these? If you should fill them it would take fifty or seventy-five little fillings to do the work. In five years' time I cut away the entire enamel from those teeth. I saw this patient last year,—it was twenty-five years ago that I did the work,—and not a single filling has been put in those teeth that I treated in that way. I am twenty-five years removing this enamel, but I save filling and save the teeth. One must be very careful in doing this work, and polishing takes a long time. But you ask, If the enamel is gone and the dentine exposed, will not the teeth decay? I answer, the enamel has little or no life, while the dentine has. By simple polishing away the enamel you get down to a substance which is unlike the dentine covered by the enamel and it becomes a substance stronger in resistance than the enamel, not only on the outside, but in every portion of the teeth. This is accomplished slowly, of course, and only with careful work. I do not wish to say anything to encourage the young man to be rash in destroying the enamel of the teeth, but I do say, have no fear or hesitation in cutting it freely when showing signs of decay.

The point I wish to impress upon you young men is that there is a dignity in every part of your work, but consider nothing beneath your attention. Preserve a sweet, wholesome, healthy mouth yourself, and help your patients to do the same. There is no portion of dental practice more important than this work of treating teeth. It is something I would emphasize and advise you to take up. It is with you young men that the future of dentistry rests. Remember that in the simplest operation in dentistry, such as amalgam filling, you should put your best efforts into the things you are attempting to make a success. Remember that the simplest operations are none of them beneath your careful attention. There are few men who care to do gutta-percha fillings, but there is no one substance that preserves the teeth better than this. The end and aim to-day seems to be to do great things to aid and further the profession. We should so teach our patients and instruct them in the care of their teeth that the necessity of our work should grow less and less year by year. It is no use for you to go into dentistry to make money. You cannot match your faculties against those of your patient, and your judgment against theirs. It is your duty, and should be your object, to instruct your patients so that they may begin and carry on the work you have commenced. You want to inspire them all with the beautiful ideal of a clean, wholesome mouth, and when you have once established that idea, they will never forget it, and will remember you all their lives, which is the highest compliment they can pay you. They will see the importance of it, and it will induce the happiest feeling.

I am sorry to have taken so much of your time, for I did not intend to talk so long. I wish to emphasize this one thought. There is no portion of your work, however humble it may be, that should not receive your best attention. Of you older men I would like to ask, Who is responsible for the prices for dental work? Dental parlor prices are, gold crowns, five dollars; silver filling, one dollar; cleaning teeth, fifty cents. If your time is worth ten dollars on a gold crown, it is certainly worth one dollar on cleaning. I had a lady say to me, not long ago, "I have been coming to you for twenty years. I was not satisfied with your charge for treating and cleaning my teeth. I want now to say to you that for twenty years you have done me the greatest service." For twenty years I was considered a robber by this educated lady.

If the spirit of helpfulness has not entered into your hearts and minds, then, indeed, have you failed to grasp the situation of your calling and the true meaning of your chosen profession as dentists. Mr. President, I thank you.

### DISCUSSION.

*Dr. Henry A. Baker,* Boston, Mass.—Mr. President, I cannot let such a subject as this and one so well dealt with go by without having something to say upon it. I know Dr. Gerrish well. I have been in practice for thirty years, and I have never listened to a fitter essay pertaining to the minor things so important to us in themselves. I endorse everything he has said, and his advice to young men is one of the most valuable lessons I have ever heard.

*Dr. A. J. Flanagan,* Springfield, Mass.—Those of us who are practising in the smaller places do not have the opportunity for making the money which those in the cities have, and in a discussion of this nature this fact should be borne in mind. As I look around and see the advancement in our profession, I sometimes come to the conclusion that the dentist gives a great deal for the amount of his fee. It is true that no patient can estimate your services for you. No one in looking into Dr. Gerrish's face could suspect that he was in the profession for the money in it. I think you will find that the public are getting all they pay for in an honest practitioner's office. One thing is true, a man reaches his level sooner or later. If he has high aspirations or low aspirations, the results will be on proportionate lines. "By their works ye shall know them."

*Dr. Mary E. Gallup,* Boston, Mass.—I have not had a great deal of experience, but find that the most trouble is caused by patients not using the tooth-brush properly. I have always used a dry brush myself, and supposed every one did. I have found only one patient in several hundred who used the tooth-brush dry, until I advised them to use it that way. I am an advocate of this method, and believe it the best way to keep the teeth mechanically clean.

*Dr. Sara P. Hooker,* Cambridge, Mass.—I am afraid that I am rather too young a member to venture any remarks on this subject. I suppose I have the same difficulties that every one else has. It is impossible to always charge for your work according to the value of it. I have been interested a little in the new method of cleaning teeth by friction and keeping the surfaces clean as a means to prevent caries, but I have not experimented with it long enough to be able to talk about it.

*Dr. Geo. A. Maxfield,* Holyoke, Mass.—I want to emphasize this one remark of Dr. Gallup's in regard to using a dry tooth-brush. It has been my custom for years, in presenting a tooth-powder, to instruct my patients to always use a dry powder on a dry brush. They are thus able to get the full benefit of the powder and to fully remove the secretions from the teeth. I have never but once before heard it submitted as being of any importance, and that was at a dental meeting in New York. While this subject is up, I want to tell you of an incident that happened in a police court at a recent trial of one of the cases for violation of our dental law. A member of this Society went on the witness-stand as an expert, and among other things he said that cleaning the teeth was not a dental operation, and was not so considered by the profession; and that he would not remove the tartar from the teeth of a patient whose teeth he was cleaning unless the patient made special request for him to do so.

*Dr. Gerrish,* Exeter, N. H.—Mr. President and friends, I thank you kindly for the gentle way in which you have handled me in discussion. I did feel audacious, as some one remarked, in coming here in this way, but I am made of the same material that you are, —clay,—endowed with a love for my work as you are, and I hope and pray that you and all of us will be the best of friends from to-day.

*President Dowsley.*—Dr. Payne expected to be here, but he has sent a paper, which will now be read by the Secretary.

### CAPSULE IMPLANTATION.

BY ROBERT EUGENE PAYNE, M.D., D.D.S., NEW YORK CITY.

Select a trephine about two-thirds the size of the normal root; cut a socket about two-thirds the depth of the normal socket. This is slightly enlarged at the bottom. The capsule should be the size of the trephine used. A seamless capsule of silver, gold, or lead is placed in position and filled with kneaded rubber.

Select a plunger nearly the size of the capsule, and by gentle pressure and lightly tapping the plunger the kneaded rubber will spread the thin capsule to every thin equality of the socket, and in this way it will be securely held in position. Into this fit a continuous gum porcelain tooth, porcelain root, and permanently cement it in the capsule. I use a two per cent. solution of cocaine before cutting the socket, and use every care to sterilize before permanently fastening the capsule in place. The operation is completed at one sitting, gives no pain in spreading the capsule, and the tissues heal kindly around it. The tooth is firm at once, it not being necessary to ligate it.

Various methods have been used to implant capsules by burnishing and otherwise, but there is no record of a seamless capsule spread in the manner above described. The operation is yet in the experimental stage, and I cannot report successes. Failure I believe to be due to an imperfect capsule, those used being too thick and rigid. I am now trying some very thin capsules, and hope to be able to report some cases at your next meeting.

The fact that Dr. Von Bergman, of Berlin, assisted by Dr. Mahl, of that city, have introduced metal splints, held by silver screws and wire to retain the fragments of the lower jaw in position after resection, that have been retained for years, leads me to believe that the capsule implantation, if carried out upon proper lines with proper material, will yield a successful capsule implantation.

At present I depend for implantation upon mature, hard, straight, natural roots, two-thirds the size of the normal root, to which I attach a porcelain crown. These roots are more lasting than any I have used during the past ten years. I have three cases, three teeth each, implantated four years ago, under very unfavorable circumstances, complicated with pyorrhœa, that are in perfect condition to-day. I will be glad to show them to any of the members that will notify me at any future date in advance, so that I may arrange a meeting.

DISCUSSION.

*Dr. W. E. Griswold,* Denver, Col.—In regard to Dr. Payne's paper, I show you here some drawings representing a system of implanting in connection with the use of my retaining springs for the support of plates. In this drawing we have an edentulous upper jaw with a section made through it in the bicuspid region. On one side is an opening made for the capsule. On the other the capsule implanted and expanded. I use a capsule made of chemically pure silver of an accurate gauge and milled on the outside. I use a trephine of exactly the same gauge, cutting down to a depth consistent with the region cut into. I place the capsule in place, measure for length, then remove it and cut off, take one of the punches going with my outfit, and punch a hole through a piece of 28-gauge plate, making a disk to go over the end about four or five lines larger than the capsule. This is soldered in place, the capsule replaced and expanded, using an oval engine burnisher. Now I use a hollow iridio-platinum tube, which just fits inside the capsule. Place inside, cut off the right length, and solder a piece of plate over its end. I notch them alike, as shown in the drawings, so that they will go back in the same place. With two or more of these implanted and prepared as shown, I take an impression of the mouth, make a model of sump, and transfer to my instrument for paralleling all my appliances, and, holding them while soldering, select a spring stud and solder it to the top. Then I use the punch, as before, and from a piece of brass about 20-gauge cut a hole, which goes over the spring. I adapt this nicely to the top, and then proceed to make my crown-cap as described in previous article. It is obvious that the soft tissues will be compressed to just the degree this brass plate is thick. The objection to any work of this kind as a means of retaining plates is the inability to implant parallel. With my system, it does not matter at what angle the capsules are placed, the attachments can always be parallel. Neither is it necessary that they be at regular intervals.

I have talked with a good many on this subject, and many agree with me that it has a probable success. Such men as Drs. Harlan and Hunt, of Chicago, have done a little in this line. Dr.

Good has also implanted some. I have one case which has been in use six years,—a case where the central incisor was extracted, the root duplicated in gold, reimplanted and expanded, and a Logan crown attached.

Dr. Prothero, of the Northwestern Dental College of Chicago, told me of a case where a friend of his met with an accident in placing the crown which necessitated extracting the root, and as the patient had to go to a reception that night, and had to have something to fill the gap, he filled the socket with amalgam and set a Logan crown in it. This at the time I saw him had, as I understood, been in use three months without any inconvenience. I do not see why we should not make as much of a success of capsule implantation as surgeons do of using silver plates in the trephining of the skull. Why should they cause irritation and be sloughed off any more than the use of silver sutures? I do not wish to be understood as endorsing the system; I simply follow Dr. Payne's idea, and show how it can be utilized as a means of support for dentures, and if it can be made a success it has great possibilities.

From a mechanical stand-point it is a success, as these capsules can be implanted so that it takes more force to remove them than you would care to exert. They require no ligation or support. They are solid when the operation is completed, and they are so small that, even if the parts will not tolerate them, no damage is permanently done to the tissues. I am having made forceps for expanding these capsules which will greatly facilitate the operation.

In a table clinic such as I gave this morning the audience gets the idea and sees the results, but does not understand the method by which these results are obtained. You get a better knowledge by following me in a description of these charts.

It consists of a series of springs, made from a new alloy of metals, discovered by myself, after some four years' experimenting, which will stand the action of the oral secretions and a heat sufficient to flow 22-carat gold solder, and at that degree not lose its high elasticity. As presented to your notice these springs are punched from this metal by a series of steel dies, brought to the form in which you see them.

I would first call your attention to that part of the system which necessitates the cutting off of crowns of teeth. To a root capped in the usual way I simply solder the spring stud, and cover this with a capsule made of platinum and iridium, carrying over it a disk, adapting very closely to the root cap, then waxing together, remove them and solder, making what I call my crown-cap, on which all superstructure is placed.

In practical work, after capping abutments, we take an impression and bite at the same time in wax, and place this on the articulator. This is merely for reference, a means whereby we can ascertain the position on the root-cap to solder the springs so they will not interfere with placing a facing in front of them, and also determine the size to use and the length we can have it, and not have it interfere with the occluding teeth.

We now take an impression of the capped roots and edentulous parts. If caps have not come away with the impression, remove and place in their respective places, before running the model; cover the inside of caps and the surface of the pins with a film of wax. When the model is made (preferably of sump, or some other non-shrinkable material) shave it perfectly smooth and level on the bottom, and if for any reason you want your bridge to go in on one side the heel or toe, first shave it so that it will tip a little that way. Now warm the caps enough to melt the wax, and remove them from the model, and after melting out all the wax replace them.

We now take the soldering jig, warm its base, put a sheet of base-plate wax over it, press the model into this softened wax, fasten with the centring pin, and clamp with the nut. Now, by observing the model on the articulator you determine where on these root-caps you can best place the stud, and not interfere with the bite, and also allow a facing to go in front of it; also the length you can have them and the right size to use. Take this stud and hold it over a Bunsen burner until it is a dull red, but do not throw it into water to cool it, as it makes it stiff again and brittle. Place it in the open end of the soldering chuck, this in the jig chuck, fastening with nut; bring it to the position you wish it to occupy on the root-cap, and observe the angle to which it must be filled to fit accurately; loosen nut and remove all these parts, and, using them as a holder, with a fine-cut gold file cut to the desired angle. Replace, tighten nut, see that No. 8 is in line with the centring pin, and tighten all nuts. Flux, place a bit of solder inside the spring, and solder, using the pointed flame held on to the root-cap until solder has flowed down and fastened all together. Now loosen nut, raise up arm, which brings the root-cap with it, loosen nut, take out chuck, remove the spring and root-cap, see that the two are firmly united, and if not, hold in a pair of pliers and complete the soldering so that each leaf of the spring is soldered solid to the root-cap. Now place a bit of vulcanizing rubber over the pin and carry down to the edge of root-band. Place the pin in a vice, so that the cap rests on the jaws, the thin edge of the band being protected by the rubber. Take the special pliers, place the capsule, and carry this over the stud. Observe the angle to which it must be filed to fit the root-cap around the base of the spring perfectly. Take special punch for the size capsule used, punch in a piece of plate large enough to cover the root-cap and extend over it, on the side the saddle comes, a hole which just fits over this capsule. Carry it to a position, and adapt perfectly to the surface of the root-cap, wax the capsule and this plate together, remove and solder heavy. Replace, then thoroughly adapt the crown-plate to the root-cap again, so that nothing can get between them. If this is a bit loose, do not worry, it is all the better, as it facilitates easy working, and you can tighten when the work is in the mouth by a very slight tap on the end.

You will proceed with each abutment in the same way; when complete, pull off the crown-caps. Replace each root-cap with the spring stud soldered to it on the roots in the mouth. If you use a saddle (this being previously made), place in position, replace the crown-caps over their studs, and as they extend over the root-caps on the side next the saddle they hold it in position; now take a bite, then select a suitable cup, punch a hole in the bottom corresponding to the shank of impression prop, place cup and prop in the mouth, see that it forces the saddle where you want it when the patient bites on the rubber cushion if the case necessitates the use of two or more of these props. While the patient is biting on them take a piece of wire and go from one to the other on the outside of the cup, twisting it around each prop and binding them all together, so that when subsequently replaced in the mouth they will go in exactly the same position. Now fill your cup with plaster, carry it to position, and have the patient bite firmly on the props, which forces your saddle into the soft tissues with exactly the same pressure that they will exert in subsequent use. Force up your tray and get an impression of all the parts in this, their true relation when in use. When hard pull out the root-caps and lay aside for polishing. Place a match or some piece of wood in the crown-caps, so that it will extend well into the model.

Now make a metal of plaster and set up on an articulator with the bite. Take a bit of sump, or some other non-shrinkable substance, and build up on this model, over the crown-caps and saddle, enough so that you can remove them all together, turn over and solder all together on the side which comes in contact with the soft tissues, replace on the model, and finish in any way desired.

There are some members of the profession, and probably many patients, who will object to the amputation of crowns, but would prefer to have shell crowns put over teeth ground down. To accommodate these people I have made these springs in a different form, but just as serviceable,—a wedged-shaped U-spring with a depression in its sides, which, when the next size is slipped over, locks the two together. These springs are made in dies, the same as the others, and in three sizes, so that one size can be used as a box for the other, and for all porcelain work. The two larger sizes are made for higher fusing.

With the outfit comes two soldering chucks, one for the small size, and one for the second size, with a milled groove in the open end to hold these springs in position for soldering to the crowns, and in perfect alignment, by placing them in the chuck of the soldering jig plate. The method of procedure is similar to that described for the spring stud. You simply take your impression of the abutments, and you wax up the inside of the crowns so that when the wax is melted they can be removed from the model. Make your model of sump, and place on the soldering jig, as before described. Select the proper size spring for the place; take this spring and pass it through the flame of a Bunsen burner until it is a dull red to anneal it, then file it to the proper length to fit the crown, upend it on a piece of gold, drawing the open ends slightly together, so that it will be a bit narrower at its base than at the upper end, and solder on the inside. Now take this and lay it, narrow side down, on another piece of gold plate, fitting it perfectly. Flux it, placing a very little solder inside again, fuse it over a Bunsen burner, soldering the two firmly together, not allowing the solder to flow up on the spring any more than can possibly be helped. Now place this on its proper soldering chuck, put this chuck in the jig chuck, and bring it to the position on the mesial or distal side of the crown you wish it to occupy. By soldering this piece of plate on the side of the spring coming in contact with the crown, it reinforces the crown at this point.

This enables it to resist the force brought to bear on it in mastication. You now take the next size, slip over it, filing to the same width and length, lay this on a piece of plate, and, springing the ends slightly together with pliers and holding in this position with them, solder on the outside, previously placing inside some whiting dissolved in alcohol, to prevent any possibility of the solder flowing inside and spoiling the perfect fit of this box for the spring. Now remove your crown from the model, which is easily done on account of the wax having been melted, and force the box over the spring stud. It will probably go over very hard and stiff; sometimes it will be necessary to use a pair of pliers to force it on and remove it; but after doing this two or three times it is put on and taken off more easily. The perfect adaptation which you get in that way is essential to perfect work. Plate G shows a model with gold crown at one end with spring soldered to it, ready to go over it and be fitted to the saddle, shown in position on the model, at the other end a stud, soldered to a root-cap, with its crown ready to slip over it. When both are in position you take an impression, as before, with sump, of these parts on the model. When hard remove and solder all three parts together, the crown, the box, and the saddle.

Now place your abutments on their respective teeth or roots, put your saddle, with its abutments soldered to it, in position, take a bite and your impression, make a model of plaster, set up on an articulator, and proceed to finish the work in any way you like.

At 10 is shown a model with abutments setting at an angle, the springs soldered on parallel, and the space between the spring and cap filled in with plate and solder, making a very solid attachment. In making this attachment, remember that the spring is altogether in compressing the outer edge, or larger diameter of the U-shaped wedge-spring; the box must be made in such a way as to compress its sides when it goes over and be rigid.

My claims for this system are, the ability to restore the contour of the soft tissues and give harmonious expression to the face, ability to avoid the display of gold, and to make the porcelain the most artistic and life-like denture known.

Secondly, even in the cheapest work, vulcanite, give your patrons something much more artistic, something held firmly in place, but removable by the patient for cleansing, and the occasional rest which the abutments need to restore to normal tone the overstrained surrounding tissues. It also divides the strain between the abutments and the edentulous parts; also to make a temporary denture which will not irritate the recently lacerated tissues, and can be replaced at any time with more permanent work, without changing the abutments or attachments. The attachments hold with more tenacity than is necessary, but the tension can be made greater or less according to the strength of the abutments.

It affords opportunity to bridge space not possible by any other system, and attachments can always be tightened, should they wear loose,—the spring studs by slight tap on the end; the U-springs by spreading a little with a pair of pliers.

DISCUSSION.

*Dr. Fred. S. Faxon,* Brockton, Mass.—To criticise a paper of this kind of course is beyond me, so far as I know anything about this class of work. It has reached a point beyond anything I have ever seen or been able to do myself. The removable bridge-work with the simple attachment between the teeth that Dr. Griswold has presented, I think entitles him to the standing in his work that Dr. Gerrish holds in the cleansing of teeth of which he has just spoken, and both are beyond criticism. In speaking of the removal bridge-work, what little experience I have had shows me that it could not be applied satisfactorily in all cases.

In regard to cutting off teeth for the stud attachment, it is not that the dentist objects to the sacrifice of the crown so much as the patient; particularly the upper cuspids. The work can be done in different ways,—by the open faced cap, although personally I do not believe in them as a rule; you can also use the Staples crown, which does not come around in front and show the gold. Many object to having a good cuspid capped on that account. Also the space can be filled by the saddle bridge with two bicuspids attached to the molar only. I have a number of cases of this kind being worn successfully. There is another class of cases in which I should have to try Dr. Griswold's method before I accepted it,—those cases where the bicuspids and molars are missing on the lower jaw, where we have only the six front teeth remaining. This class of cases has always been very unsatisfactory to handle in my practice, that is to get a firm eating surface in the back part of the mouth where the condition is such as I have spoken of. In these cases, and they are the most difficult ones to treat, I have never been able to make nor have I ever seen any dovetailing attachment but what was either very complicated, looked bungling, or showed too much gold. For these cases I have found nothing so simple, firm, and satisfactory, all points considered, as a telescope cap attachment. The only gold showing is our cap on each side, while with Dr. Griswold's method the cap on abutment teeth and also the attachment teeth on the removal piece must be a heavily tipped facing, thus showing practically two gold teeth on each side. My experience has been so satisfactory with the telescope cap in this class of cases, that, although I shall take advantage of an opportunity to try this method of Dr. Griswold's, I hardly feel that I shall be converted. As I said before, it is not my purpose to criticise this method, as I heartily endorse it in every particular, except the class of cases last referred to.

*Dr. Griswold.*—In reply to Dr. Faxon, with the first tooth, the one coming next to the gold crown, there is a difficulty of covering the box and not displaying gold, but the ingenious dentist will generally overcome that. We use, wherever possible, rubber teeth, and, at the most, display only the top of the box, which looks like a gold filling in the tooth, especially if it is built up a bit with gold. In very short teeth we might have to use a grinding surface of gold. By the use of a diamond disk you can generally overcome the difficulty without much trouble. This system is not supposed to be universal in its application any more than any other; it has its place, and that is where the teeth are fairly long.

*Dr. W. H. Stowe,* Boston, Mass.—I have only seen this work of Dr. Griswold's within the past few days, but not being very busy, I looked over the circular I received very carefully, and it struck me that it was a very good thing; and a few days after that I called upon the doctor and he showed me his models and explained more fully, and after a careful consideration we decided that it was an excellent thing. I became so much interested in it that arrangements have been made whereby we will act as his agents, and will be able to furnish the dentists with these appliances complete or any parts, as they may wish. We will also be able to teach them how to use the appliance and enable them to do this work themselves. Before Dr. Griswold leaves the city I shall make myself thoroughly familiar and conversant with it and I have no doubt that it will help out in a great many difficult cases.

*Dr. Griswold.*—In reply to Dr. Baker, my experience is very limited. The first case was an emergency case, the only thing my patient would think of letting me try, as he would not have a bridge or wear a plate, but this and Dr. Payne's work led me to think of and try and perfect the work from a mechanical point of view. It was purely experimental, but are we not warranted in making the experiment, when, as I said, we are not doing any permanent injury, even if it is a failure? In conclusion, I do not know the name of the gentleman who said he did not wish to criticise me, but what I want is criticism, and it is only through it that you are able to perfect anything. There is no question but that the U-springs can be boxed so that they will hold any denture without posterior attachments so they will not drop or raise up at the heel. I have two cases in the mouth of Dr. Fillebrown's niece which have been worn for nearly six months with comfort and satisfaction. That is one great advantage of the work, the ability to hold that class of cases firmly.

I thank you, gentlemen, for your courteous attention.

Waldo E. Boardman, D.M.D.
*Editor Massachusetts Dental Society.*

